# Blood Distribution of SARS-CoV-2 Lipid Nanoparticle mRNA Vaccine in Humans

**DOI:** 10.1021/acsnano.4c11652

**Published:** 2024-09-19

**Authors:** Stephen J. Kent, Shiyao Li, Thakshila H. Amarasena, Arnold Reynaldi, Wen Shi Lee, Michael G. Leeming, David H. O’Connor, Julie Nguyen, Helen E. Kent, Frank Caruso, Jennifer A. Juno, Adam K. Wheatley, Miles P. Davenport, Yi Ju

**Affiliations:** † Department of Microbiology and Immunology, Peter Doherty Institute for Infection and Immunity, The University of Melbourne, Melbourne, Victoria 3000, Australia; ‡ Melbourne Sexual Health Centre and Department of Infectious Diseases, Alfred Hospital and Central Clinical School, Monash University, Melbourne, Victoria 3000, Australia; § School of Science, RMIT University, Melbourne, Victoria 3000, Australia; ∥ Infection Analytics Program, Kirby Institute for Infection and Immunity, University of New South Wales, Sydney, New South Wales 2052, Australia; ⊥ Melbourne Mass Spectrometry and Proteomics Facility, The Bio21 Molecular Science and Biotechnology Institute, The University of Melbourne, Parkville, Victoria 3010, Australia; # Department of Chemical Engineering, The University of Melbourne, Melbourne, Victoria 3000, Australia

**Keywords:** PEGylated lipid nanoparticle, COVID-19, kinetics of mRNA, immunoglobulins, biomolecular coronas, particle–immune cell interactions

## Abstract

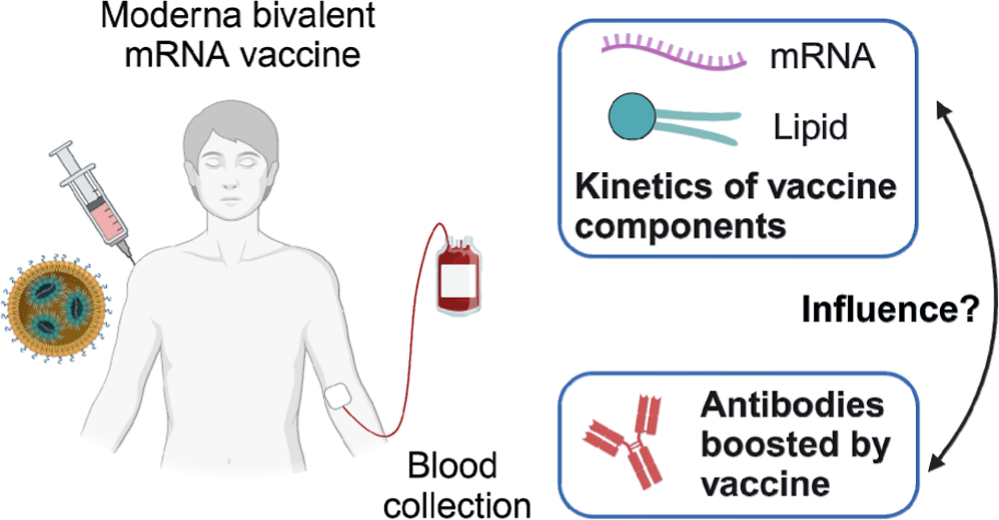

Lipid nanoparticle mRNA vaccines are an exciting but emerging technology used in humans. There is limited understanding of the factors that influence their biodistribution and immunogenicity. Antibodies to poly(ethylene glycol) (PEG), which is on the surface of the lipid nanoparticle, are detectable in humans and boosted by human mRNA vaccination. We hypothesized that PEG-specific antibodies could increase the clearance of mRNA vaccines. To test this, we developed methods to quantify both the vaccine mRNA and ionizable lipid in frequent serial blood samples from 19 subjects receiving Moderna SPIKEVAX mRNA booster immunization. Both the vaccine mRNA and ionizable lipid peaked in blood 1–2 days post vaccination (median peak level 0.19 and 3.22 ng mL^–1^, respectively). The vaccine mRNA was detectable and quantifiable up to 14–15 days postvaccination in 37% of subjects. We measured the proportion of vaccine mRNA that was relatively intact in blood over time and found that the decay kinetics of the intact mRNA and ionizable lipid were identical, suggesting the intact lipid nanoparticle recirculates in blood. However, the decay rates of mRNA and ionizable lipids did not correlate with baseline levels of PEG-specific antibodies. Interestingly, the magnitude of mRNA and ionizable lipid detected in blood did correlate with the boost in the level of PEG antibodies. Furthermore, the ability of a subject’s monocytes to phagocytose lipid nanoparticles was inversely related to the rise in PEG antibodies. This suggests that the circulation of mRNA lipid nanoparticles into the blood and their clearance by phagocytes influence the PEG immunogenicity of the mRNA vaccines. Overall, this work defines the pharmacokinetics of lipid nanoparticle mRNA vaccine components in human blood after intramuscular injection and the factors that influence these processes. These insights should be valuable in improving the future safety and efficacy of lipid nanoparticle mRNA vaccines and therapeutics.

Lipid nanoparticle mRNA vaccines have revolutionized vaccinology and saved countless lives during the COVID-19 pandemic.^1^ Lipid nanoparticle mRNA vaccines typically contain 5 materials—mRNA, an ionizable lipid, a poly(ethylene glycol) (PEG) –lipid, a helper lipid, and cholesterol. Although the vaccines are delivered intramuscularly (IM) and act primarily in the draining lymph node, recent studies have suggested that at least small amounts of the mRNA vaccines may distribute in humans more widely than originally anticipated. A primary cross-sectional study detected mRNA in blood for up to 15 days after mRNA vaccination.^2^ Full-length or traces of the SARS-CoV-2 spike mRNA vaccine sequences were identified in plasma up to 28 days postvaccination via RNA sequencing.^3^ Low levels of the vaccine mRNA were detected in breast milk up to 45 h postvaccination.^4,5^ An autopsy study of people dying incidentally after vaccination found mRNA in tissues (axillary lymph nodes and heart) up to 30 days after vaccination.^6^ Presumably, the mRNA reached breast milk and tissues following circulation in the blood. Despite evidence in animals and humans that mRNA can be detected in blood after vaccination,^2,7^ studies of the pharmacokinetics of mRNA lipid nanoparticle components in blood in humans are lacking.

Vaccines that contain nonhuman materials other than the vaccine antigens, such as adenovirus vectors, can induce immune responses to those products, known as antivector responses. If strong enough, such antivector responses can clear the vaccine more rapidly, limiting the immunogenicity of booster vaccinations or potentially causing other unintended effects.^8,9^ We recently found that mRNA vaccines can boost PEG-specific antibodies in humans,^10^ confirmed by multiple groups.^11−14^ PEG is however relatively weakly immunogenic and the PEG-specific antibodies remained relatively low (end point binding titer generally <10^3^) after 2 mRNA vaccinations and did not influence the immunogenicity of the vaccines.^10^ Nonetheless, the long-term consequences of boosting PEG-specific antibodies after multiple mRNA vaccinations are unknown.^15^ In particular, even the modest boost in anti-PEG antibodies resulted in detectable increases in the ability of human blood monocytes to phagocytose nanomaterials containing PEG in vitro.^10^ This may be a greater concern for the clearance of PEGylated nanomaterials administered intravenously in contrast to IM-delivered mRNA vaccines, which primarily act at the draining lymph node rather than circulating more widely.

We hypothesized that we could quantify the decay kinetics of small amounts of lipid nanoparticle mRNA vaccines that spill into the blood and that the decay rates would be influenced by levels of PEG antibody levels. We further hypothesized that vaccine mRNA immunogenicity and the capacity of monocytes to phagocytose lipid nanoparticles might also be influenced by lipid nanoparticle levels or PEG antibodies in the blood. To evaluate this, we studied IM-delivered mRNA vaccines in a cohort of 19 humans through serially sampling plasma early after vaccination and developing methods to quantify the vaccine mRNA and ionizable lipid by PCR and mass spectrometry, respectively (Figure 1a).

**Figure 1:**
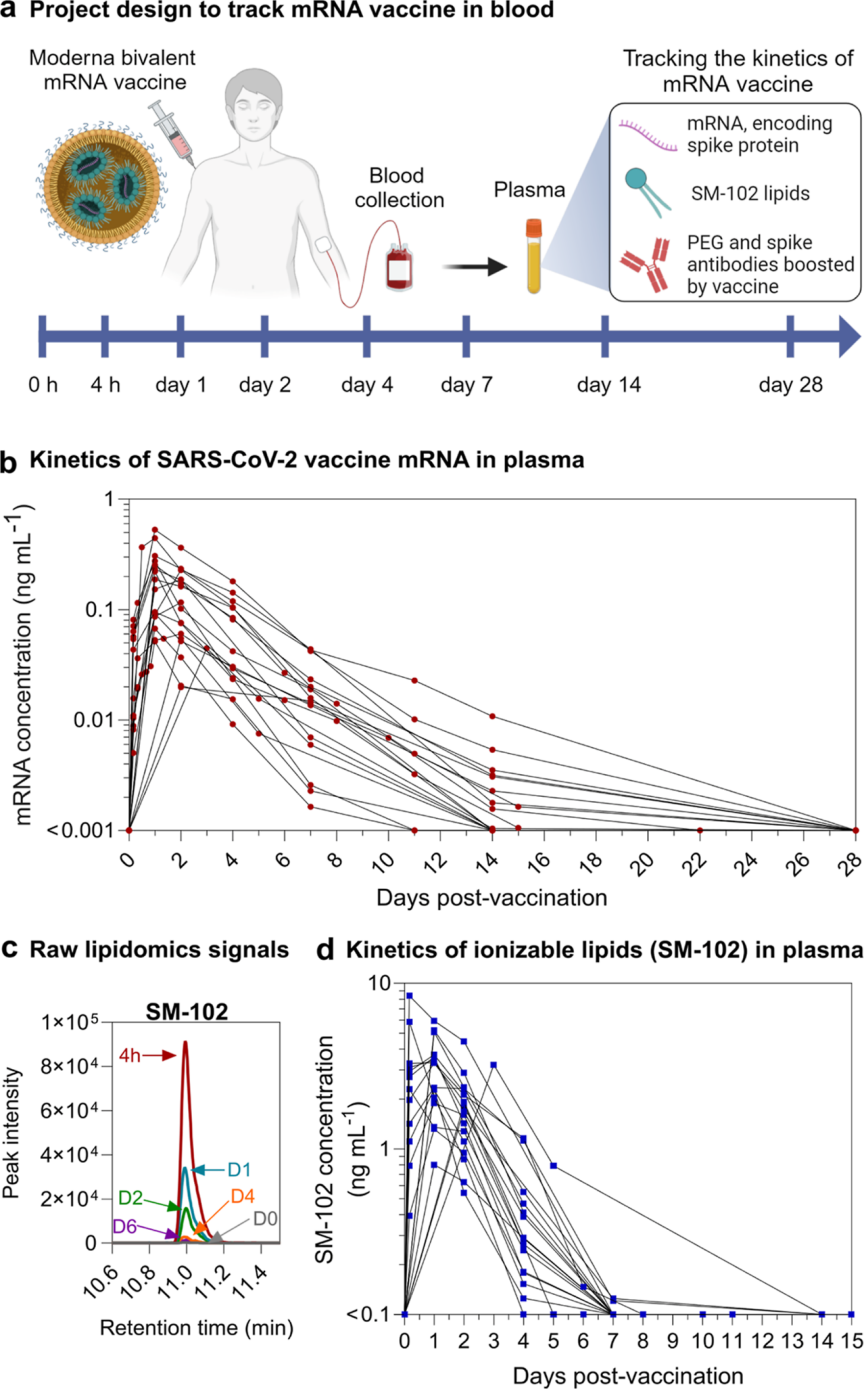
In vivo kinetics of mRNA and ionizable lipid from the SPIKEVAX SARS-CoV-2 mRNA vaccine in human blood. (a) Schematic illustration of the project design to track the kinetics of mRNA vaccine in human blood of 19 healthy subjects who received one dose of Moderna SPIKEVAX COVID-19 bivalent mRNA vaccine (see Table S1 for subject information). Plasma samples were collected at prevaccination (day 0) and at a median of 8 (range 4–14) other time points between 4 h and 28 days postvaccination. Created with BioRender.com. (b) Longitudinal vaccine mRNA concentrations in the plasma of the 19 subjects. To improve readability, the detailed mRNA kinetics within the first 24 h of vaccination is shown in Figure S2. (c) Representative image showing the peak intensity of SM-102 signals in a set of plasma samples from a subject at day 0–6 postvaccination determined by liquid chromatograph mass spectrometry. (d) Longitudinal SM-102 ionizable lipid concentrations in the plasma of the 19 subjects. The concentrations of vaccine mRNA and SM-102 ionizable lipid were calculated based on the linear standard curves and raw data in Figure S1.

## Results and Discussion

### Human Subjects

We studied 19 subjects receiving bivalent Moderna SPIKEVAX booster immunization. The subjects ranged from 24 to 70 (mean 42) years old with 63% females and had received 3–4 (median 3) doses of monovalent COVID-19 vaccines before receiving the bivalent mRNA vaccine (details of subjects and vaccines in Table S1). A total of 156 blood samples (median 9, range 5–15 samples per subject) were serially collected prevaccination and from 4 h to 28 days postvaccination. Plasma (with EDTA or heparin anticoagulation) was stored within 4 h at −80 °C and PBMCs were isolated by Ficoll separation and stored in liquid nitrogen.

### Kinetics of COVID-19 Vaccine mRNA in Human Blood

A reverse transcription droplet digital PCR (ddPCR) method was developed to detect and quantify the COVID-19 vaccine mRNA (see the Methods section for details). All prevaccination blood samples (which were a minimum of 139 days after any previous mRNA vaccination, Table S1) were negative for COVID-19 vaccine mRNA (Figures 1b and S1c). Vaccine mRNA was detected in the plasma samples of all 19 bivalent booster vaccine subjects at 4 h postvaccination (range 6.5–112 mRNA copies μL^–1^, equivalent to 0.005–0.081 ng mL^–1^), peaked at 1–2 (mean 1.3) days post vaccination (at peak levels of up to 731 mRNA copies μL^–1^, equivalent to 0.529 ng mL^–1^), and subsequently displayed log-linear decay kinetics (Figures 1b, S1c, and S2). The mRNA kinetics of individual subjects are shown in Figure S3. Small amounts of mRNA (0.001–0.01 ng mL^–1^) remained above the lower limit of quantification (LLOQ) in 37% of Moderna vaccine subjects’ plasma (7 out of 19 subjects) at day 14 or 15 postvaccination.

### Kinetics of COVID-19 Vaccine Ionizable Lipids in Human Blood

SM-102 is a nonhuman ionizable lipid used to interact with mRNA in the lipid nanoparticle formulation of the Moderna SPIKEVAX mRNA vaccine. Since SM-102 has a structure that is distinct from endogenous human lipids, a mass spectrometry-based lipidomics method was developed to detect and quantify SM-102 lipids in human plasma (Figure 1c, see the Methods section for details). SM-102 background signal was determined in plasma without SM-102 (collected prevaccination), and levels were detected above background (range 0.39–8.39 ng mL^–1^) in the plasma of all 19 Moderna vaccine subjects at 4 h postvaccination (Figures 1d, S1d, and S3). SM-102 levels peaked at 4 h to 2 days (mean 1.1 day) postvaccination (median 3.22 ng mL^–1^) and subsequently showed log-linear decay kinetics. The SM-102 signals remained significantly above the background at day 4 postvaccination (up to 1.16 ng mL^–1^) and approached background levels by day 7 postvaccination (up to 0.12 ng mL^–1^).

### Degradation on Vaccine mRNA In Vivo

**Figure 2:**
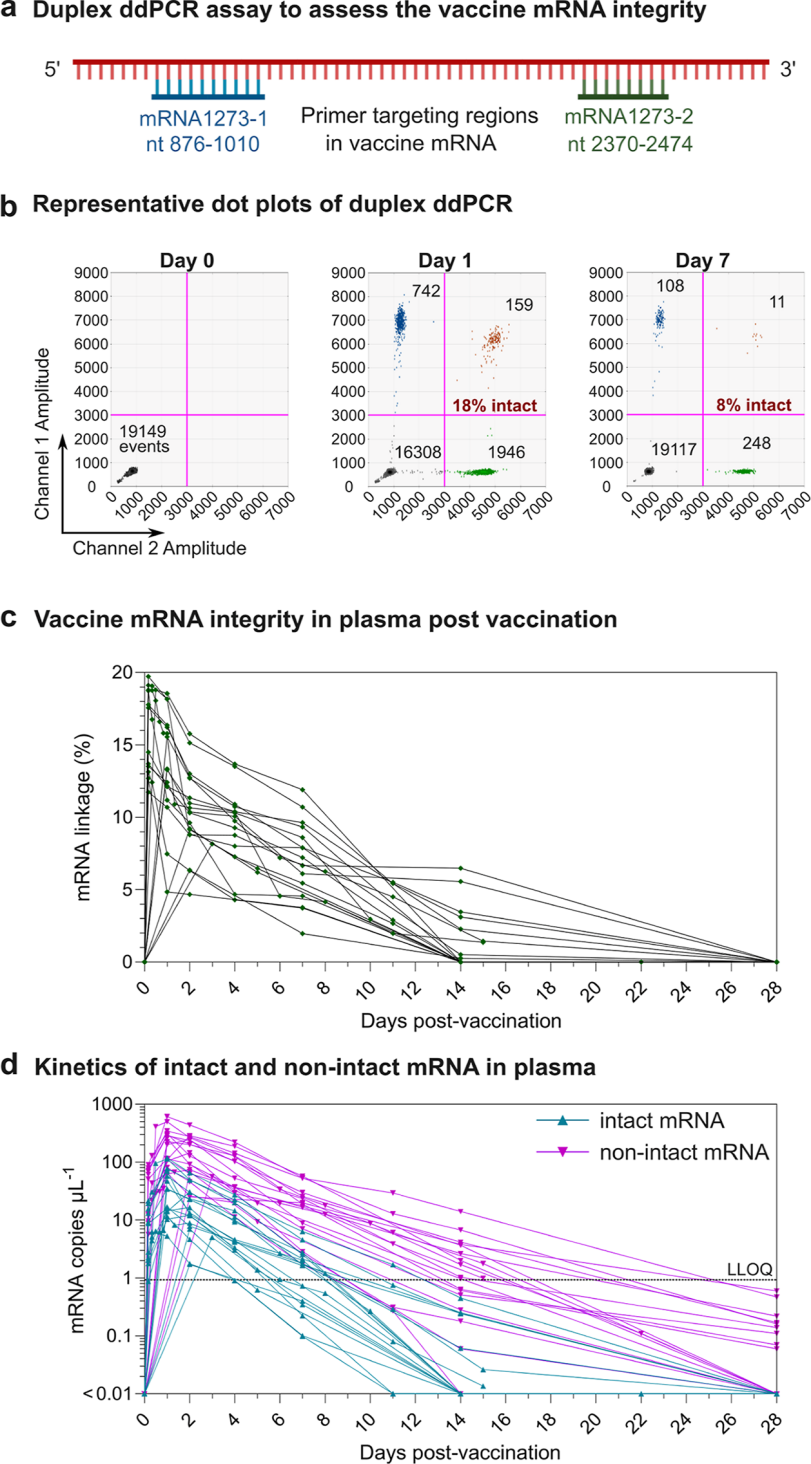
Integrity of vaccine mRNA in plasma after vaccination with SPIKEVAX SARS-CoV-2 mRNA vaccine. (a) Schematic illustration of a duplex ddPCR assay using a two-primer set targeting two regions (mRNA1273-1 nt 876–1010 and mRNA1273-2 nt 2370–2474) of the mRNA1273 sequence. (b) Representative dot plot profiles of FAM-labeled mRNA1273-1 primer and probe (channel 1, amplitude) and HEX-labeled mRNA1273-2 primer and probe (channel 2, amplitude) at day 0 (left panel), 1 (middle panel), and 7 (right panel) postvaccination. Droplets emitting 2D signals were separated into four groups (gray: double negative for mRNA1273-1 and mRNA1273-2; blue: positive for mRNA1273-1, negative for mRNA1273-2; green: positive for mRNA1273-2, negative for mRNA1273-1; and orange: double positive for both mRNA1273-1 and mRNA1273-2). (c) Vaccine mRNA integrity in plasma of 19 subjects postvaccination. Vaccine mRNA integrity was assessed by mRNA linkage (%), which was expressed as the estimated percent of linked molecules (correcting for the frequency of random association of probes). The number of droplets in each single or double positive group was derived by QX Manager software. (d) Longitudinal intact and nonintact mRNA levels in the plasma of the 19 subjects before and after vaccination. The intact mRNA levels were calculated by multiplying the mRNA linkage (%) by the total mRNA levels detected in plasma. The LLOQ (shown as a dashed line) is determined based on the linear standard curves of vaccine mRNA at 0.001 ng mL^–1^ (Figure S1a). To improve readability, the detailed mRNA integrity kinetics within the first 24 h of vaccination is shown in Figure S4.

mRNA is labile in blood at 37 °C when not protected by a lipid nanoparticle.^16^ Total vaccine mRNA (which is >2000 bp) was detected in plasma in the studies above using a short 113 bp ddPCR reaction, which would detect both intact and some degraded mRNA. To assess the levels of intact and degraded vaccine mRNA in plasma, we adapted a linkage ddPCR technique developed by Hanna et al., which analyzes whether both 3′ and 5′ fragments of the vaccine mRNA can be amplified in a single droplet (Figure 2a; see the Methods section for details).^4^ Amplifying both fragments (double positive events) suggests that the mRNA is relatively intact (i.e., spans both PCR reactions), which can be quantified mathematically by comparing single- and double-positive event levels. Using this assay, we were able to track the degradation kinetics of vaccine mRNA in vivo. A representative read-out of the linkage ddPCR assay for 3 time points in one subject is shown in Figure 2b. Only a small proportion (<20%) of vaccine mRNA was found to be intact and relatively stable within 4–24 h postvaccination across 19 subjects (Figure S4a). However, the proportion of intact mRNA consistently and gradually decreased over time (Figure 2c). It is worth noting that the linkage ddPCR assay we used depends on the relative efficiency of the two PCR reactions and the specific manner in which the mRNA degrades. Other assays assessing mRNA integrity (such as those performed by vaccine manufacturers) may reveal different absolute results. However, the comparative levels over time and decay rates using the same assay should be accurate and indeed showed a steady decline in all subjects.

Measuring the level of total vaccine mRNA and the proportion of intact vaccine mRNA allowed us to calculate the levels of intact and nonintact mRNA over time (Figures 2d, S3, and S4b). Both intact and nonintact mRNA exhibited log-linear decay kinetics. The levels of intact mRNA remained above the LLOQ up to day 11 postvaccination in one subject but dropped below the LLOQ in all 19 subjects by 14 days postvaccination. The kinetics of nonintact mRNA appeared similar to those of total mRNA (Figure S3) as the majority of mRNA detected in the plasma is nonintact.

**Figure 3:**
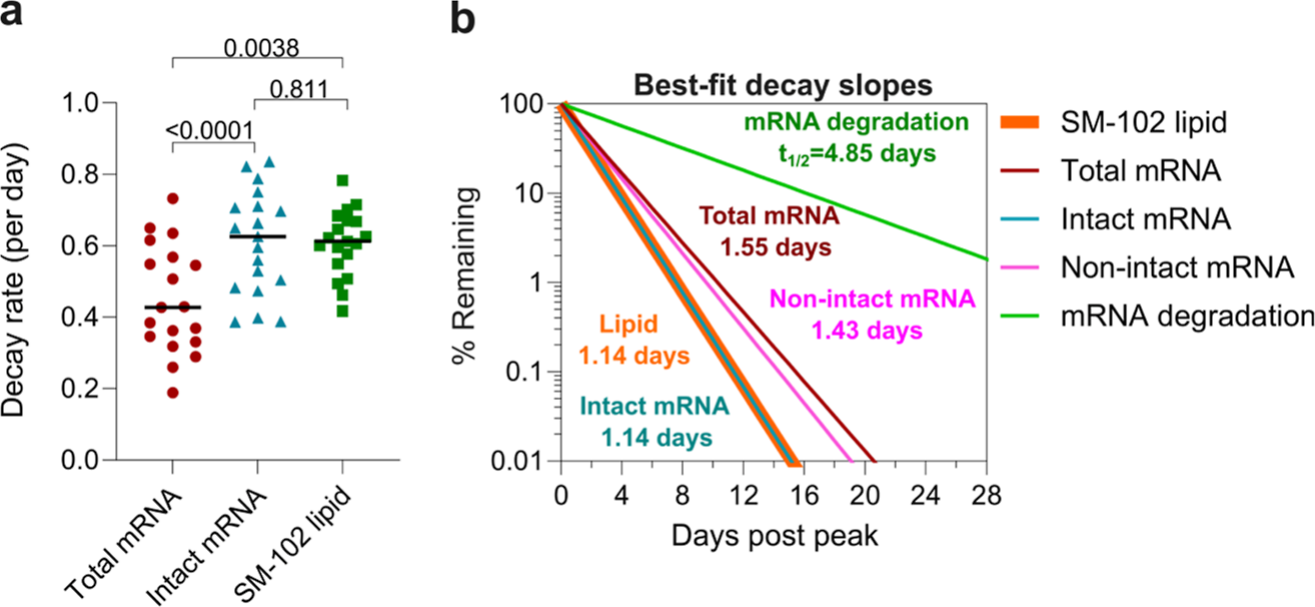
Dynamics of vaccine mRNA and SM-102 lipids in the plasma. (a) Comparison of decay rate between total mRNA, intact mRNA, and SM-102 lipid. Statistics assessed by the likelihood ratio test. (b) Best-fit decay slops of SM-102 lipids, total mRNA, intact mRNA, nonintact mRNA, and the rate of degradation of intact mRNA. The responses at the first time point (the peak time) for each parameter are set to 100%, and the change (%) over time and half-life are shown. As the decay slopes of SM-102 lipid and intact mRNA overlap, the curve of the SM-102 lipid slope was plotted with higher thickness than that of the intact mRNA slope to improve readability.

The detection of both intact vaccine mRNA and the SM-102 lipid in the blood suggests that the lipid nanoparticles containing both materials may be circulating in the blood. If this were the case, the decay kinetics of both elements would be similar. Indeed, the decay rate (mean 0.608 day^–1^) and half-life (1.14 days) of intact mRNA was essentially identical to that of the ionizable lipid (mean decay rate 0.607 day^–1^, half-life 1.14 days, Figure 3). Nonintact mRNA had a slower decay than intact mRNA (half-life of 1.43 vs 1.14 days, Figure 3b). The reason for the longer circulation time of nonintact mRNA is unclear, but is likely to be related with the properties of the lipid nanoparticles when loaded with small fragments of mRNA. The slow degradation of the mRNA despite circulating in blood in vivo at 37 °C (half-life 4.85 days, Figure 3b), and the identical decay rate of intact mRNA and the ionizable lipid, suggests that the mRNA was largely protected in circulation within the lipid nanoparticle.

### Expansion of Anti-PEG Antibodies

**Figure 4:**
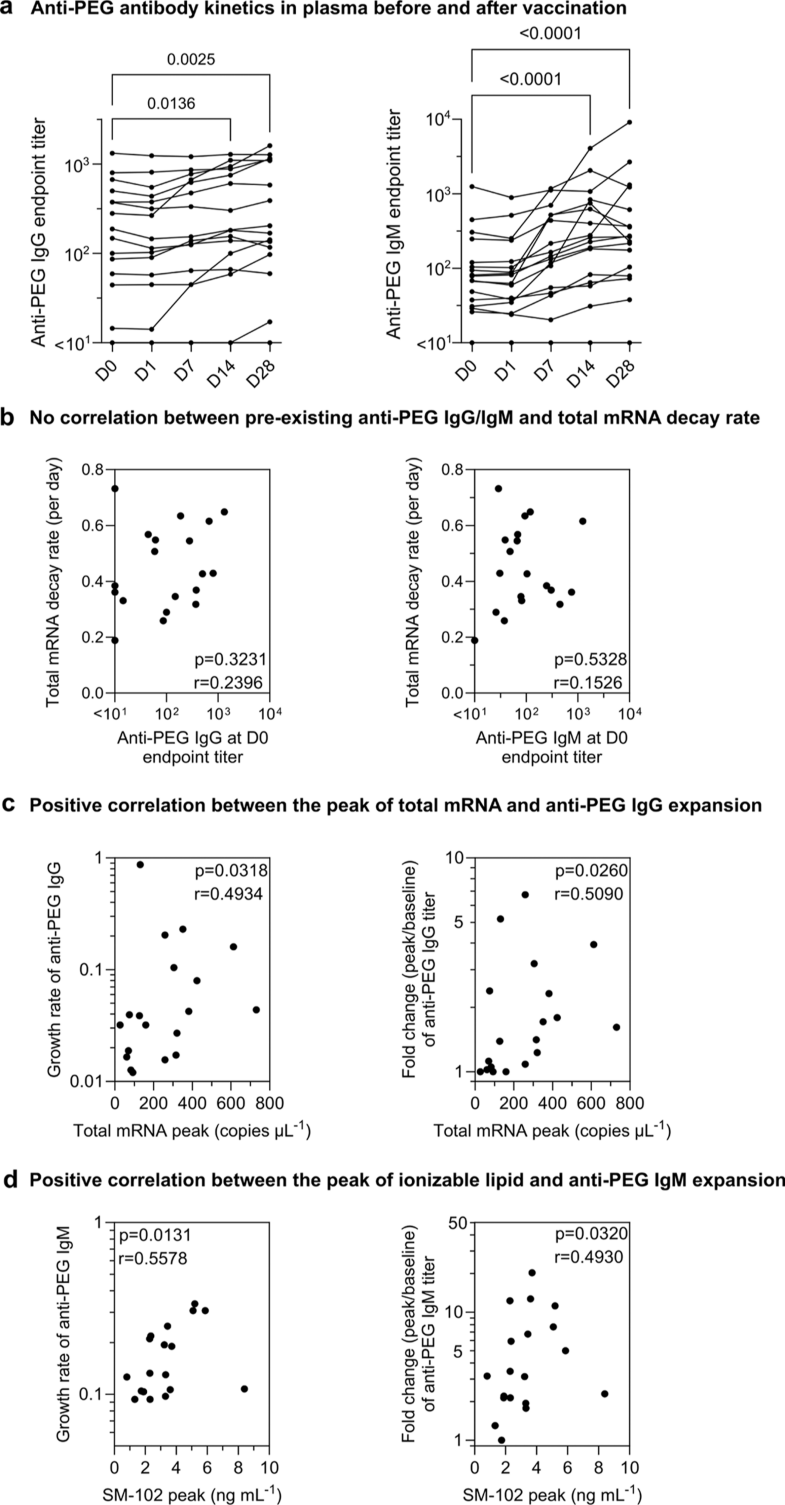
Kinetics of anti-PEG antibody and their correlation with vaccine mRNA kinetics. (a) Longitudinal anti-PEG IgG and IgM titers in the plasma before and after IM inoculation of SPIKEVAX SARS-CoV-2 mRNA vaccine. Statistics assessed by nonparametric Friedman’s test with Dunn’s multiple comparisons test (*n* = 17 as 17 subjects have all five time points). (b) No significant correlation between pre-existing anti-PEG antibody titers and total mRNA decay rate across the 19 subjects. (c) Significant positive correlation between the peak levels of total mRNA in plasma and anti-PEG IgG expansion (growth rate/fold change) across the 19 subjects. (d) Significant positive correlation between the peak levels of ionizable lipid (SM-102) and anti-PEG IgM expansion (growth rate or fold change) across the 19 subjects. Statistics in (a–c) were assessed by Spearman correlation analysis (*n* = 19).

PEG-specific IgG and IgM antibodies in human plasma were quantified by ELISA using our established method.^10^ Anti-PEG IgG and IgM were detectable (end point binding titer >1:10) prevaccination in the plasma of 15 and 18 of the 19 subjects, ranging in titer from 1:15 to 1:1321 and 1:26 to 1:1247, respectively (Figure 4a). Following immunization, an increase in PEG-specific IgG and IgM was observed with a mean fold change of 1.4 (range 1–6.7) and 4.6 (range 1–20.3) at day 28 postvaccination, respectively. This was less than the fold change of PEG IgG (13.1, range 1–70.9) and IgM (68.5, range 0.9–377.1) following a 2-dose primary Moderna mRNA-1273 vaccination reported in our previous study.^10^ Longitudinal analyses showed that PEG-specific antibodies were boosted in a time-dependent manner with a significant increase observed from day 14 postvaccination (Figure 4a). The higher boost in anti-PEG IgM compared to IgG induced by mRNA vaccines is consistent with our previous study and those of others.^10,13,14^ This may be related to the lack of T-cell help and poor isotype switching. However, the potential mechanism could be more complex and may depend on the specificity of the regional population as opposite trends (higher boost in anti-PEG IgG compared to IgM) have also been observed in other studies.^11,12^

We hypothesized that the clearance of mRNA lipid nanoparticles might be influenced by the levels of anti-PEG antibodies in the blood through PEG antibody-induced opsonization, a phenomenon called “accelerated blood clearance”.^17^ Accelerated clearance of PEGylated nanoparticles by anti-PEG antibodies has been observed in murine studies.^18,19^ However, most murine studies use IV, rather than IM, administration of PEGylated nanoparticles or proteins to induce PEG antibodies or detect clearance. The relevance of these IV administration routes in rats or mice to IM administration of mRNA vaccines in humans is unclear.^20^

We found that the titer of pre-existing anti-PEG antibodies was not significantly correlated with the decay rate of mRNA or ionizable lipids (Figures 4b and S5). The relatively low levels of mRNA in blood, the relatively uniform decay rates across the 19 subjects, and the generally modest levels of anti-PEG antibodies were potential factors associated with the lack of a correlation between anti-PEG antibodies and mRNA clearance. Interestingly, the peak levels of mRNA and ionizable lipids in the blood postvaccination positively correlated with subsequent anti-PEG IgG and IgM expansion, respectively (Figure 4c,d), suggesting that the boost in anti-PEG antibodies may be influenced by the amounts of lipid nanoparticle mRNA vaccines distributed in the blood. We noted that the correlations drawn from anti-PEG IgG expansion need further validation with larger cohort studies, as the majority of subjects experienced only a modest boost in anti-PEG IgG postvaccination.

If anti-PEG antibodies had a major interaction with mRNA lipid nanoparticles in the blood, we might expect anti-PEG antibodies to complex with the vaccine-derived nanoparticles and evade detection in our ELISA early after vaccination. However, we found only a very small reduction in anti-PEG IgG antibodies at day 1 postvaccination compared to prevaccination (Figure S6). This suggests that the amounts of lipid nanoparticles distributed into the blood were not high enough to have a major impact on the levels of anti-PEG antibodies.

### Expansion of Anti-Spike and Neutralizing Antibodies

**Figure 5:**
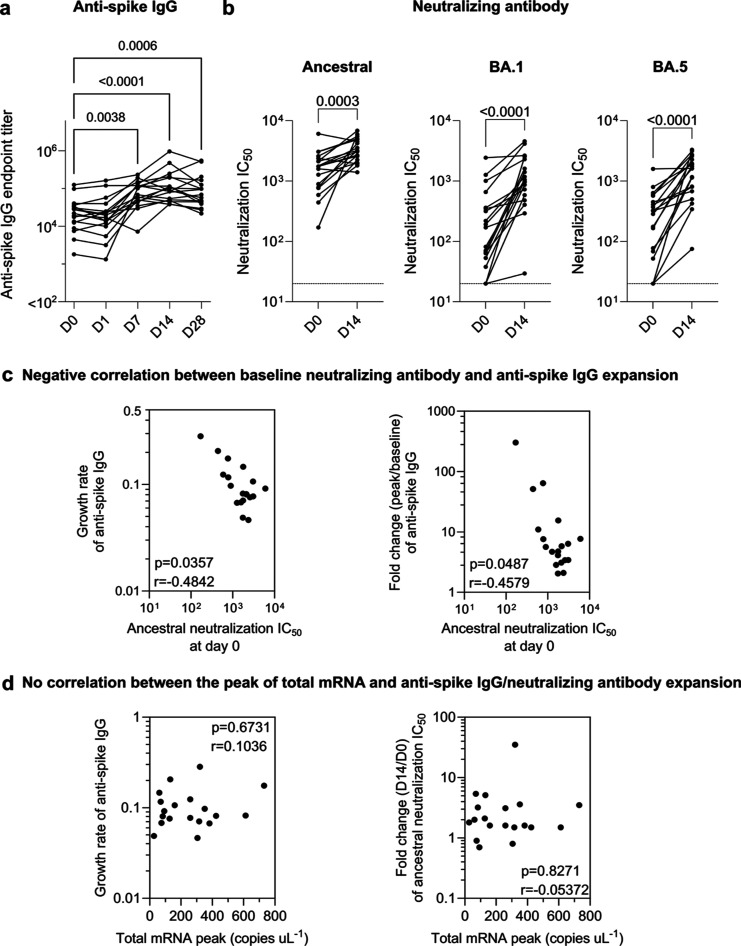
Kinetics of antispike IgG and neutralizing antibody. (a) Longitudinal antispike IgG titers in the plasma of the Moderna bivalent mRNA vaccinee cohort (Table S1). Statistics assessed by nonparametric Friedman’s test with Dunn’s multiple comparisons test (*n* = 17 as 17 subjects have all five time points). (b) Comparing live SARS-COV-2 neutralization titer (inhibitory concentration 50, IC_50_) before vaccination (day 0) and postvaccination at day 14 of the Moderna bivalent mRNA vaccinee cohort. The limit of detection at a titer of 1:20 is shown in the dashed line. Statistics assessed by nonparametric Wilcoxon’s matched-pairs signed rank test (*n* = 19). (c) Significant negative correlation between baseline neutralizing antibodies and antispike IgG expansion (growth rate or fold change). (d) The peak levels of total vaccine mRNA in the plasma do not influence the expansion of antispike IgG and neutralizing antibody. Statistics in (c,d) were assessed by Spearman correlation analysis.

The vaccine mRNA immunogenicity is reflected by the boost of antispike binding and SARS-CoV-2 neutralizing antibodies (Figure 5a,b), which were evaluated by ELISA end point dilution and a live virus neutralization assay, respectively. All the 19 subjects have received 3 or 4 doses of COVID-19 monovalent vaccination with an interval of 354 (range 139–496) days before the bivalent booster (Table S1). Following the bivalent booster, the spike-specific IgG end point titer increased with a mean fold change of 21.3 (range 1.4–302.4) at day 28 postvaccination in all 19 subjects. As expected, the increase of spike binding antibody titer positively correlated with the boost of neutralizing antibody titer (Figure S7). The neutralizing antibody titer against ancestral (original), BA.1 (Omicron subvariant), and BA.5 (Omicron subvariant) strains of SARS-CoV-2 were boosted with a mean fold change of 4.0 (range 0.7–34.9), 15.5 (range 1.1–126.8), and 15.3 (range 1.0–164.7), respectively. The fold change of ancestral neutralizing antibodies was lower than BA.1 and BA.5 as all the 19 subjects have already developed a high level of ancestral neutralizing antibodies (mean IC_50_ 1838, range 170–34919) prior to the booster vaccine. This is also consistent with the negative correlation between the baseline ancestral neutralizing antibody titer and the spike IgG binding titer increase (Figure 5c). We assessed whether vaccine mRNA immunogenicity was influenced by the levels of mRNA in the blood. However, no correlation was observed between mRNA levels in the blood and the expansion of spike-binding IgG or neutralizing antibodies (Figure 5d). This suggests that circulating mRNA is not a primary driver of antispike responses, which is consistent with the primary role of the draining lymph node in stimulating immunity.

### Influence of Monocyte Phagocytosis of Lipid Nanoparticles

**Figure 6:**
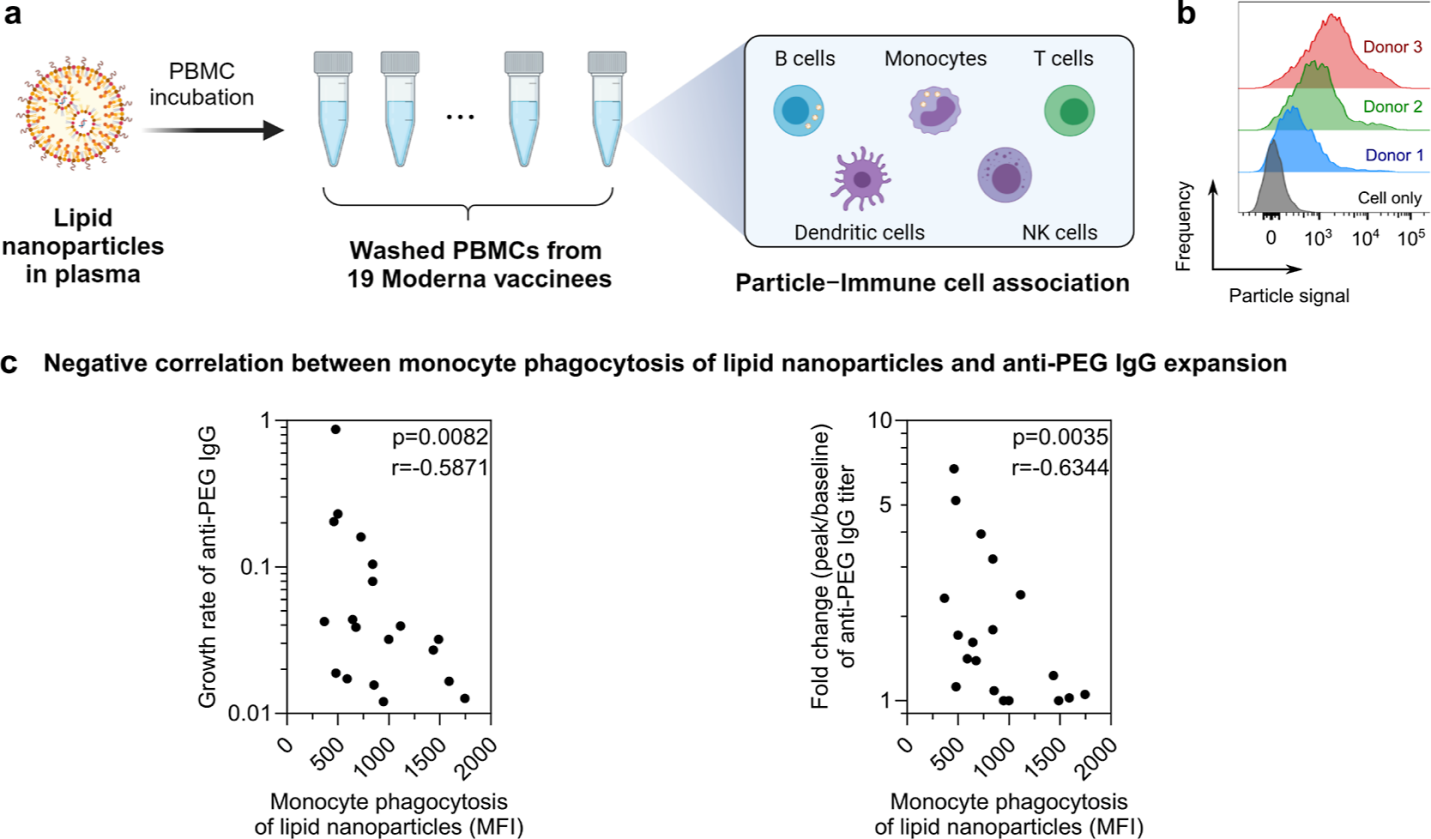
Human blood assay to assess lipid nanoparticle–immune cell interactions. (a) Schematic illustration of the in vitro assay to assess the person-specific cellular interactions of primary immune cells with lipid nanoparticles. PBMCs (collected from the 19 subjects before receiving the Moderna SPIKEVAX bivalent vaccination) were washed by centrifugation with serum-free media multiple times to completely remove plasma. Lipid nanoparticles were preincubated with human plasma from 1 subject and then incubated with PBMCs from the 19 subjects in serum-free media for 1 h at 37 °C, followed by phenotyping cells with antibody cocktails and analysis by flow cytometry. Created with BioRender.com. (b) Flow cytometry histograms represent the monocyte phagocytosis of lipid nanoparticles after incubating with PBMCs of 3 different subjects. Cell-only control groups show the respective cell populations without particles in the incubation media. (c) Significant negative correlation between monocyte phagocytosis of lipid nanoparticles (median fluorescence intensity, MFI) and anti-PEG IgG expansion (growth rate or fold change). Statistics were assessed by Spearman correlation analysis.

The positive correlations between vaccine components (mRNA and ionizable lipids) in blood and anti-PEG antibody expansion observed in Figure 4c,d suggest that the boost in anti-PEG antibodies may be influenced by the amount of mRNA lipid nanoparticles circulating in the blood. When mRNA lipid nanoparticles are first distributed into the bloodstream, they are subjected to phagocytosis by blood monocytes and neutrophils, a process that is person-specific, as demonstrated in our previous study.^21^ We hypothesized that the capacity of blood phagocytes to take up lipid nanoparticles could influence the systemic bioavailability of the lipid nanoparticles and, in turn, modulate the PEG immunogenicity of mRNA vaccines. Herein, we modified a previously developed human blood nanoparticle association assay^21^ to explore the person-specific cellular interactions with primary immune cells, including lymphocytes and monocytes. Peripheral blood mononuclear cells (PBMCs) were available prevaccination from 19 of the vaccinees. The cells were washed in serum-free media before incubation with lipid nanoparticles for 1 h at 37 °C (Figure 6a). The primary immune cells were subsequently labeled with fluorescent antibody cocktails and analyzed by flow cytometry to quantify the cellular association of lipid nanoparticles (see details in Methods, gating strategy shown in Figure S8). The lipid nanoparticles were formulated using the same molar composition of lipids as clinically used Moderna (SPIKEVAX) lipid nanoparticle formulation and were preincubated with human plasma from one subject to form biomolecular coronas before incubating with PBMCs from 19 subjects. The lipid nanoparticles displayed a donor-dependent association with monocytes and B cells with minimal association with T cells, natural killer cells, and dendritic cells (Figures 6b and S9). Our previous study has demonstrated that particles are likely internalized by monocytes, while they remain exclusively bound to the cell membrane of B cells.^21^ The association of nanoparticles with B cells is likely mediated by complement receptors on B cells as previous studies have shown that heat inactivation of plasma, which destroys complement activity, inhibits this interaction.^22^ We observed a negative correlation between the ability of monocytes to interact with lipid nanoparticles and the increase in the anti-PEG IgG titer (Figures 6c and S10). In contrast, the association of lipid nanoparticles with other cell types showed no correlation with the expansion of PEG antibodies (Figure S11).

## Conclusions

We found that both vaccine mRNA and ionizable lipids can be detected from plasma at 4 h following the bivalent Moderna SPIKEVAX booster, peaking at around day 1 in blood and showing a subsequent log-linear decay profile. We further showed the slow degradation of intact vaccine mRNA in blood. The similar kinetics of intact mRNA and the ionizable lipid in blood and the slow degradation of the mRNA suggest that mRNA lipid nanoparticles remain intact and travel from injection sites or lymph nodes into the bloodstream within 4 h postvaccination. The rapid dissemination of mRNA lipid nanoparticles in blood found in our study is consistent with the recent findings on the detection of mRNA in breast milk at 3–45 h postvaccination.^4^ We detected low levels of mRNA in plasma above LLOQ up to 14–15 days after vaccination. This is consistent with recent cross-sectional and autopsy studies.^2,3,6^

We initially hypothesized that the decay rate of mRNA (lipid nanoparticles) would be influenced by the levels of anti-PEG antibodies as many animal studies have demonstrated the phenomenon of accelerated blood clearance.^20^ However, we did not observe such a correlation (Figure 4b). The relatively low levels of anti-PEG antibodies in the blood and relatively uniform levels of mRNA decay across the subjects suggest that mRNA decay may be more of an intrinsic feature of humans and less susceptible to external factors such as PEG antibodies. We speculate that humans with much higher levels of PEG antibodies, such as those receiving PEGylated therapeutics intravenously, may clear lipid nanoparticle mRNA vaccines more quickly.

We did, however, observe that the peak amounts of mRNA and ionizable lipid (lipid nanoparticles) detected in the blood had a positive correlation with the subsequent expansion of anti-PEG IgG and IgM (Figure 4c,d). We also observed a negative correlation between the level of in vitro monocyte phagocytosis of lipid nanoparticles and anti-PEG IgG expansion (Figure 6c). These findings suggest that the amounts of mRNA lipid nanoparticles that remain in the blood (and free of phagocytosis) may influence the PEG antibody immunogenicity in humans. This phenomenon only affected PEG immunogenicity as the mRNA levels in the blood were not significantly correlated with the expansion of spike-binding IgG or neutralizing antibodies (Figure 5d). This is consistent with PEG being expressed on the surface of the lipid nanoparticle, whereas the spike protein, the target of neutralization and spike binding IgG, is only expressed following the mRNA transfection of cells. Larger cohort studies and animal studies are required to confirm the causal relationship between the levels of mRNA lipid nanoparticles in blood and the expansion of anti-PEG antibodies.

Additional factors could influence the biodistribution and immunogenicity of mRNA lipid vaccines. Humans display a diverse range of plasma lipids and proteins. Similar differences are likely to be found in lymphatic fluids, which vaccines must traverse. The proteins and lipids bind to mRNA lipid nanoparticles in either lymph or plasma, forming biomolecular corona,^23,24^ which could influence the uptake and immunogenicity of the vaccines.^25−28^ We previously showed that clinically relevant drug-loaded liposomes displayed a large variance in immune cell association in human blood, depending on the compositions of person-specific biomolecular coronas.^21^ The formation of biomolecular corona on drug-loaded liposomes in vivo in human blood has been previously described.^29^ The composition of biomolecular coronas has been shown to influence the fate and functionality of lipid nanoparticles.^30,31^ An analysis of the in vivo or ex vivo corona formation on mRNA vaccines could help further characterize mRNA vaccine distribution and immunogenicity.

Overall, our study provides important information on the kinetics of mRNA lipid nanoparticle vaccines in human blood in vivo and their impact on vaccine and PEG immunogenicity. Enhancing our understanding on the biodistribution of mRNA lipid nanoparticles in humans should ultimately help improve the safety and efficacy of mRNA vaccines and therapeutics.

## Methods

### Ethics Statement

The study protocols were approved by the University of Melbourne Human Research and Ethics Committee (approvals no. 2056689), and all associated procedures were carried out in accordance with the approved guidelines. All participants provided written informed consent in accordance with the Declaration of Helsinki.

### Participant Recruitment and Sample Collection

Participants were recruited through contacts with the investigators and were invited to provide serial blood samples. We recruited 19 participants who received a bivalent Moderna SPIKEVAX booster immunization. Participants’ characteristics are collated in Table S1. For all participants, whole blood was collected with sodium heparin or ethylenediaminetetraacetic acid (EDTA) anticoagulant or using a serum-separating tube. Plasma and serum were collected and stored at −80 °C, and PBMCs were isolated via Ficoll–Paque separation, cryopreserved in 10% dimethyl sulfoxide/fetal calf serum (FCS) and stored in liquid nitrogen.

### Quantification of COVID-19 Vaccine mRNA

COVID-19 vaccine mRNA was quantified by reverse transcription ddPCR. Total RNA was isolated from 140 μL of plasma (EDTA as anticoagulant) using the QIAmp RNA Extraction Kit (cat. no. 52906 Qiagen). RNA (10 μL) was subjected to reverse transcription, using Superscript III following the manufacturer’s recommendation (Invitrogen). Primers and probe were designed and synthesized (Integrated DNA Technologies) based on the putative sequence of Moderna COVID bivalent (SPIKEVAX Bivalent Original/Omicron BA.4–5). These primer and probe sets are specific to the respective codon-modified vaccine mRNA sequence and do not amplify the wild-type S-gene (see sequences in Table S2).

ddPCR was performed by using a QX200 Droplet Digital PCR system (Bio-Rad). The ddPCR reaction mixture consisted of 2× ddPCR Supermix (12 μL) for probes (no dUTP, Bio-Rad, cat #1863024), cDNA (5 μL), and primers/probes mix (5 μL) to a final volume of 24 μL. Droplet generation was achieved using a DG8 Droplet Generator Cartridge (cat #1864008, Bio-Rad) and Droplet Generator (Bio-Rad). Amplification was carried out on a C1000 Touch thermal cycle (Bio-Rad) using a thermal profile beginning with 95 °C for 10 min, followed by 40 amplification cycles of 94 °C for 30 s, 60 °C for 60 s, and ending with 98 °C for 10 min (ramp rate 2 °C/s for each step). After PCR, the plate was subsequently read on a QX200 droplet reader (Bio-Rad), and data were analyzed with QuantaSoft 1.7.4 software.

Preparations incorporating prevaccination plasma samples were used as negative controls where there was no vaccine mRNA. Vaccine mRNA was spiked into the negative human plasma (collected prevaccination), followed by cDNA reaction, 10-fold series dilution in nuclease-free water, and ddPCR reaction to set the positive droplet threshold and generate a linear PCR standard curve. The copy number of the vaccine mRNA in the PCR reaction was used to derive the copy number per milliliter of plasma. The linear standard curve (Figure S1a) of vaccine mRNA PCR data was derived in GraphPad Prism 10 with relative weighting (weighting by 1/*y*^2^). The concentrations of vaccine mRNA were calculated based on the linear standard curve (Figure S1a).

### Quantification of Ionizable Lipids

Ionizable lipids SM-102 were detected and quantified via targeted mass spectrometry. Lipids were extracted from 50 μL of diluted plasma (90%, a mixture of 5 μL phosphate-buffered saline (PBS) and 45 μL neat plasma) using 500 μL of 1-butanol/methanol (1:1, v/v), followed by vortexing for 10 s and sonication for 60 min in a sonication water bath at 20 °C. The samples of the linear SM-102 standard curve were prepared by mixing 5 μL of lipid nanoparticles (with known SM-102 concentration in PBS) into 45 μL of plasma (collected prevaccination) at a final concentration of SM-102 from 0.1 to 12.5 ng mL^–1^, followed by the same lipid extraction procedure. All the plasma and standard samples were subsequently centrifuged at 16,000*g* for 20 min, and 100 μL of the supernatant was transferred to glass vials before mass spectrometry analysis.

Samples were analyzed using a Shimadzu 8050 triple quadrupole mass spectrometer coupled to a Shimadzu Nexera X2 liquid chromatography unit. Components of plasma and calibrations samples were separated using an Agilent RRHD Eclipse Plus C18 column (2.1 × 1000 mm, 1.8 μm; Agilent Technologies, USA) over a 15 min gradient using 6:4 water/acetonitrile containing 10 mM ammonium acetate and 5 μM medronic acid as mobile phase A and 9:1 isopropanol/acetonitrile containing 10 mM ammonium acetate as mobile phase B. The solvent gradient was as follows [time (min), B (%)]: [0, 5], [2, 5], [10, 90], [12, 90], [12.5, 5], [15, 5]. The LC solvent flow rate was 0.3 mL min^–1^,and the eluent was diverted to waste for the first 5 min of the analysis. The autosampler chamber and column oven were maintained at 10 and 40 °C, respectively, throughout the analysis.

Compounds eluting from the column were introduced into the gas phase by electrospray ionization. The nebulizing, heating gas flow rate was set to 2 L min^–1^, and both the heating and drying gas flow rates were set to 10 L min^–1^. The interface temperature was set to 350 °C and the interface voltage was 4 kV. SM-102 was analyzed in positive ion mode using a multiple reaction monitoring (MRM) strategy (Table S3). Collision energies and parameters for SM-102 detection were optimized using the in-built Shimadzu LabSolutions MRM optimization tool.

Raw mass spectrometry data were analyzed using Skyline (v23.1). Analytes were quantified by integration of the peak areas of the summed MRM transitions, and the linear standard curve (Figure S1b) was constructed by weighting of standard sample intensities by 1/*x*^2^. The concentrations of SM-102 ionizable lipids were calculated based on the linear standard curve (Figure S1b).

### Linkage ddPCR to Measure the Relative Levels of Intact mRNA

We employed the methods of Hanna et al. to perform two simultaneous ddPCR reactions, linkage duplex ddPCR at either end of the spike mRNA using probes with different fluorescence (FAM or HEX).^4^ If both PCR tests are positive within a single droplet, it suggests that the droplet contains mRNA that spans both regions, indicating that the mRNA is relatively intact. If the droplet contains mRNA for only one end of the mRNA, it suggests that the mRNA is relatively degraded. By quantifying the proportion of droplets in which both assays yield amplification, samples containing intact vaccine mRNA (positive linkage) can be distinguished from samples containing fragmented mRNA. The percent of linkage of each sample was expressed as the percentage of linked molecules in relation to the total molecules detected. Linkage number was calculated by QuantaSoft 1.7.4 software, which determined the excess of double-positive droplets over the expected due to random colocalization of unlinked targets. The following formula is used to calculate the % of linkage
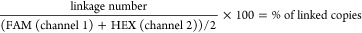


The two sets of probes/primers are shown in Table S4, and the methods are as described in Hanna et al.,^4^ which is similar to the ddPCR conditions described for mRNA quantification above.

### Quantification of Anti-PEG Antibody

The ELISA to detect anti-PEG IgG and IgM was conducted using a previously developed method.^10^ Briefly, the eight-arm PEG-NH_2_ (40 kDa, 200 μg mL^–1^, JenKem Technology, USA) in PBS was coated onto MaxiSorp 96-well plates (Nunc, Denmark) for 18 h at 4 °C, followed by washing with PBS four times. Plates were blocked with 5% (w/v) skim milk powder in PBS for 22 h, followed by adding serially diluted human plasma in 5% skim milk in duplicate for 1 h at 22 °C. Plates were washed with 0.1% 3-[(3-cholamidopropyl)-dimethylammonio]-1-propanesulfonate (CHAPS, Sigma-Aldrich, USA)/PBS buffer twice and PBS four times prior to addition of an HRP-conjugated antihuman IgG (Dako Agilent, USA) at 1:20,000 dilution or HRP-conjugated antihuman IgM (Jackson ImmunoResearch Laboratories, USA) at 1:10,000 for 1 h at 22 °C. Plates were washed as above and then developed using 3,3′,5,5′-tetramethylbenzidine (TMB) liquid substrate (Sigma-Aldrich, USA). The reaction was stopped with 0.16 M H_2_SO_4_ and read at 450 nm. End point titers were calculated as the reciprocal plasma dilution giving signal 2× background using a fitted curve (4-parameter log regression) and reported as a mean of duplicates. Background was detected by adding the diluted plasma samples (at a 1:10 dilution in 5% skim milk) to the non-PEG-coated wells, followed by the same ELISA procedure.

### Quantification of Anti-Spike IgG

Plasma antibody binding to the SARS-CoV-2 ancestral spike protein was tested by ELISA. 96-well Maxisorp plates were coated overnight at 4 °C with 2 μg mL^–1^ recombinant spike protein (Hexapro^32^). After blocking with 1% FCS in PBS, duplicate wells of 4-fold serially diluted plasma (1:100–1:1,638,400) were added and incubated for 2 h at room temperature. Bound antibody was detected using 1:20,000 dilution of HRP-conjugated antihuman IgG (Agilent, P021402-5, USA). Plates were then developed using the TMB substrate, the reaction was stopped using sulfuric acid, and the plates were read at 450 nm. Plates were washed 3–6 times with PBS and 0.05% Tween-20 following incubations. End point titers were calculated using GraphPad Prism as the reciprocal serum dilution that gave an OD reading of 2× background using a fitted curve (4 parameter log regression).

### SARS-CoV-2 Virus Propagation and Titration

Ancestral SARS-CoV-2 (hCoV-19/Australia/VIC01/2020) isolate was grown in Vero cells in serum-free DMEM with 1 μg mL^–1^ TPCK trypsin while Omicron BA.1 (hCoV-19/Australia/NSW-RPAH-1933/2021) and BA.5 (hCoV-19/Australia/VIC61194/2022) strains were grown in Calu3 cells in DMEM with 2% FCS. Cell culture supernatants containing infectious virus were harvested on day 3 for VIC01 and day 4 for Omicron strains, clarified via centrifugation, filtered through a 0.45 μM cellulose acetate filter, and stored at −80 °C.

Infectivity of virus stocks was then determined by titration on HAT-24 cells (a clone of transduced HEK293T cells stably expressing human ACE2 and TMPRSS2^33,34^). In a 96-well flat bottom plate, virus stocks were serially diluted 5-fold (1:5–1:78,125) in DMEM with 5% FCS, added with 30,000 freshly trypsinized HAT-24 cells per well and incubated at 37 °C. After 46 h, 10 μL of alamarBlue Cell Viability Reagent (Thermo Fisher) was added into each well and incubated at 37 °C for 1 h. The reaction was then stopped with 1% SDS and read on a FLUOstar Omega plate reader (excitation wavelength 560 nm and emission wavelength 590 nm). The relative fluorescent units (RFUs) measured were used to calculate the % viability (“sample” ÷ “no virus control” × 100), which was then plotted as a sigmoidal dose–response curve on GraphPad Prism to obtain the virus dilution that induces 50% cell death (50% infectious dose; ID_50_). Each virus was titrated in quintuplicate in 3–5 independent experiments to obtain mean ID_50_ values.

### SARS-CoV-2 Microneutralization Assay with Viability Dye Readout

Plasma neutralization activity was measured against live SARS-CoV-2 (ancestral, BA.1, and BA.5), as described previously.^34^ In 96-well flat bottom plates, heat-inactivated plasma samples were diluted 3-fold (1:20–1:43,740) in duplicate and incubated with SARS-CoV-2 virus at a final concentration of 2 × ID_50_ at 37 °C for 1 h. Next, 30,000 freshly trypsinized HAT-24 cells in DMEM with 5% FCS were added and incubated at 37 °C. “Cells only” and “virus + cells” controls were included to represent 0% and 100% infectivity, respectively. After 46 h, 10 μL of alamarBlue Cell Viability Reagent (Thermo Fisher) was added into each well and incubated at 37 °C for 1 h. The reaction was then stopped with 1% SDS and read on a FLUOstar Omega plate reader (excitation wavelength 560 nm and emission wavelength 590 nm). The RFUs measured were used to calculate %neutralization with the following formula: (“sample” – “virus + cells”) ÷ (“cells only” – “virus + cells”) × 100. Inhibitory concentration 50 (IC_50_) values were determined using four-parameter nonlinear regression in GraphPad Prism with curve fits constrained to have a minimum of 0% and maximum of 100% neutralization.

### Lipid Nanoparticle Preparation and Characterization

Lipid nanoparticles were formulated with heptadecan-9-yl 8-[2-hydroxyethyl-(6-oxo-6-undecoxyhexyl)amino]octanoate (SM-102, MedChemExpress, USA), distearoylphosphatidylcholine (DSPC, Avanti Polar Lipids, USA), cholesterol (Sigma-Aldrich, USA), and 1,2-dimyristoyl-*rac*-*glycero*-3-methoxypolyethylene glycol-2000 (PEG2000-DMG) (MedChemExpress, USA) with the same molar composition of lipids used in the US FDA-approved Moderna SPIKEVAX formulation, in addition to 0.1 mol % dioctadecyl-3,3,3,3-tetramethylindodicarbocyanine (DiD, Thermo Fisher Scientific, USA), using the NanoAssemblr platform (Precision NanoSystems, Canada). Particles were loaded with a nonimmunogenic nucleic acid cargo (firefly luciferase plasmid DNA, PlasmidFactory GmbH & Co. KG, Germany) in order to regulate lipid packing and particle size. The concentration of encapsulated pDNA in lipid nanoparticles (98 μg mL^–1^) was determined using the Quant-iT PicoGreen dsDNA Assay kit with an encapsulation efficiency of >97%. The size (68 ± 18 nm) and polydispersity index (0.08) of the lipid nanoparticles were determined by dynamic light scattering analysis performed on a Zetasizer Nano-ZS instrument (Malvern Instruments, UK). The zeta-potential (−10 ± 3 mV) of the lipid nanoparticles were determined by using a Zetasizer Nano-ZS (Malvern Instruments, UK) where particles were dispersed at pH 7.4 in phosphate buffer (5 mM).

### Blood Assay to Determine Lipid Nanoparticle Association with Human Immune Cells

The frozen PBMCs from the 19 subjects (collected before receiving the Moderna SPIKEVAX vaccination) were thawed at 37 °C, washed twice with serum-free RPMI 1640 medium, and counted with a Cell-DYN Emerald analyzer. The DiD-labeled nanoparticles (3.4 μg based on pDNA loading) were preincubated in 680 μL of plasma (collected from 1 subject prevaccination) at 37 °C for 1 h to allow the formation of biomolecular coronas around the nanoparticles. The lipid nanoparticles (0.05 μg) in the presence of plasma were subsequently incubated with PBMCs (5 × 10^5^) from 19 subjects in the serum-free RPMI 1640 medium (100 μL) at a particle concentration of 0.45 μg mL^–1^ for 1 h at 37 °C. After incubation, PBMCs were washed with PBS (4 mL, 500 g, 7 min) and stained for phenotypic markers in PBS at 4 °C for 1 h using titrated concentration of antibodies against CD45 V500 (H130, BD), CD19 BV650 (HIB19, BioLegend), CD14 APC-H7 (MΦP9, BD), CD3 AF700 (SP34-2, BD), CD56 PE (B159, BD), lineage-1 (Lin-1) cocktail FITC (BD), and HLA-DR PerCP-Cy5.5 (G46-6, BD). Unbound antibodies were removed by washing twice with cold (4 °C) PBS containing 0.5% (w/v) BSA and 2 mM EDTA (4 mL, 500 g, 7 min). Cells were fixed with 1% (w/v) formaldehyde in PBS and directly analyzed by flow cytometry (LSRFortessa, BD Bioscience). The data were processed using FlowJo V10 with the gating strategy shown in Figure S8.

### Estimating the Decay Rates

The decay rate of mRNA and lipid in plasma was estimated by fitting a linear mixed effect model as a function of days postpeak and response type (comparing total mRNA, intact mRNA, and SM-102 lipid). The likelihood ratio test was used to determine if the decay rate is different with respect to the response type. We fitted the model to log-transformed data of various response variables (assuming exponential decay), and we censored the data from below (left-censoring) if it was less than the lower limit of quantitation. The model was fitted by using the *lmec* library in R (v4.2.1), using the maximum likelihood algorithm.

The activation time and growth rate of PEG IgG, PEG IgM, and spike IgM following vaccination were estimated as previously described.^35^ A piecewise model was used in which the immune response *y* for subject *i* at time *y*_*i*_ can be written as
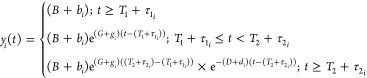


The model has 5 parameters: *B*, *G*, *T*_1_, *D*, and *T*_2_. For a period before *T*_1_, we assumed a constant baseline value *B* for the immune response (which is higher than or at the background level). After the activation time, *T*_1_, the immune response will grow at a rate of *G* until *T*_2_. From *T*_2_, the immune response will decay at a rate of *D*. For each subject *i*, the parameters were taken from a normal distribution, with each parameter having its own mean (fixed effect). A diagonal random effect structure was used where we assumed there was no correlation within the random effects. The model was fitted to the log-transformed data values, with a constant error model distributed around zero with a standard deviation σ. To account for the values less than the limit of detection, a censored mixed effect regression was used to fit the model. Model fitting was performed by using Monolix2023R1.

### Statistical Analysis

Associations between mRNA, anti-PEG antibodies, antispike IgG, neutralizing antibodies, and cellular association of lipid nanoparticles were assessed using nonparametric Spearman correlation in GraphPad Prism 10. Decay rates of total mRNA, intact mRNA, and SM-102 lipid were compared by the likelihood ratio test in R (v4.2.1). Pair-wise comparison of neutralization IC_50_ between D0 and D14 and anti-PEG antibody between D0 and D1 were assessed by nonparametric Wilcoxon’s matched-pairs signed rank test in GraphPad Prism 10. Longitudinal comparison in anti-PEG antibodies, antispike IgG, and SM-102 signals were derived by nonparametric Friedman’s test with Dunn’s multiple comparisons test in GraphPad Prism 10.

## Supporting Information Available

Demographic information on donors who received the Moderna SPIKEVAX SARS-CoV-2 bivalent mRNA vaccine; sequences of primer and probe designed for PCR assay; optimized MRM parameters for SM-102 detection; standard curves, raw PCR and lipidomics data showing the kinetics of mRNA and ionizable lipids in plasma; kinetics and integrity of vaccine mRNA within first 24 h postvaccination; individual subject plots showing the kinetics of SM-102 ionizable lipid, total mRNA, intact mRNA, and nonintact mRNA; correlation between pre-existing anti-PEG antibodies and the decay rate of ionizable lipid; anti-PEG antibody levels prevaccination and day 1 postvaccination; correlation between the boost of spike IgG and the boost of neutralizing antibody; gating strategy used to identify immune cell populations from human PBMCs; influence of person-specific PBMCs on lipid nanoparticle–immune cell interactions; correlation between monocyte phagocytosis of lipid nanoparticles and anti-PEG IgG expansion; and correlation between B cell/T cell/NK cell/dendritic cell association (MFI) and anti-PEG IgG expansion (PDF)

## Notes

A version of this manuscript was posted to the preprint server *medRxiv* on July 27, 2024 as the following: Kent, S. J.; Li, S.; Amarasena, T. H.; Reynaldi, A.; Lee, W. S.; Leeming, M. G.; O’Connor, D. H.; Nguyen, J.; Kent, H. E.; Caruso, F.; Juno, J. A.; Wheatley, A. K.; Davenport, M. P.; Ju, Y. Blood Distribution of SARS-CoV-2 Lipid Nanoparticle mRNA Vaccine in Humans. *medRxiv* 2024; 10.1101/2024.07.25.24311039.
